# Anomalous origin of the left circumflex coronary artery: is surgery required? A case report

**DOI:** 10.1093/ehjcr/ytad641

**Published:** 2023-12-23

**Authors:** William Murray, Ros Lavery, Jim O’Brien

**Affiliations:** Department of Cardiology, Mater Private Network, Eccles Street, Dublin D07WKW8, Ireland; Department of Cardiology, Mater Private Network, Eccles Street, Dublin D07WKW8, Ireland; Department of Cardiology, Mater Private Network, Eccles Street, Dublin D07WKW8, Ireland

**Keywords:** Case report, Anomalous left circumflex, Main pulmonary artery, ALCxAPA Ventricular Tachycardia

## Abstract

**Background:**

Deviations from usual coronary artery anatomy are well documented. The left circumflex artery (LCx) arising from the pulmonary artery is an example of one such deviation which is rarely seen. We present the case of a 26-year-old male with this coronary artery distribution presenting with an episode of ventricular flutter with late gadolinium enhancement and pluri-morphological ventricular arrhythmias.

**Case summary:**

A 26-year-old male with a history of cardiac surgery presented to his local hospital with an episode of symptomatic broad-complex tachycardia (BCT). It failed to revert to sinus rhythm following intravenous beta-blockers and amiodarone and required external cardioversion. Subsequently, the patient developed a aspiration pneumonia requiring ICU admission, after which he was transferred to our institute for ongoing cardiac management. Cardiac computed tomography CTA and coronary angiography revealed that the LCx was found to originate from the pulmonary artery. He underwent insertion of a subcutaneous pacemaker and was subsequently discharged. Despite the potential for steal syndrome of viable coronary territories. Multidisciplinary team discussion determined him to be fit for conservative management and not for surgical correction of his anomalous coronary artery anatomy.

**Discussion:**

Aberrant coronary artery anatomy can lead to diverse outcomes for patients in terms of both morbidity and mortality. The need for surgery in these situations varies on a case-by-case basis and little research exists to guide decision-making for healthcare professionals. As such there is a need for further study both to guide treatment and to ensure high-quality outcomes for patients with this condition.

**Learning points**

- Anomalous coronary artery distribution, although rare can have a significant effect on a patient’s ability to tolerate increased myocardial workload.
- Surgical data on outcomes following and indications for re-implantation of anomalous coronary arteries is limited and requires further research.
- Computed tomographicangiography and cardiac magnetic resonance (CMR) play a key role in diagnosing anomalous coronary artery distributions.

## Introduction

Deviations from usual coronary artery anatomy are well documented.^1^ The left circumflex artery (LCx) arising from the pulmonary artery is an example of one such deviation which is rarely seen with one systematic review finding only 46 documented cases to date.^1^ This case of a 26-year-old male with a surgically created coronary artery distribution of this nature presenting with an episode of ventricular flutter with associated transmural myocardial scar and pluri-morphological ventricular arrhythmias highlights the importance of identifying patients with this anatomy early and effectively coordinating subspecialties within cardiology to maximally improve patient outcomes.

## Summary figure

**Figure:**
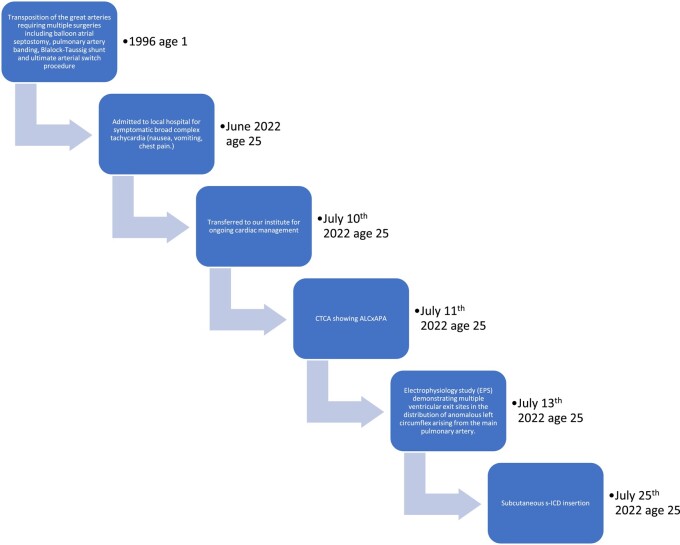


## Case presentation

Our patient initially presented to his local hospital with nausea, dizziness, palpitations, and one episode of vomiting which started 22 h previously. The symptoms began shortly after an episode of cocaine, alcohol, and caffeine consumption of unknown quantity. The patient had no chronic medical conditions and was not taking any regular medications. The patient had no known drug allergies and did not have a significant smoking history.

**Figure 1:**
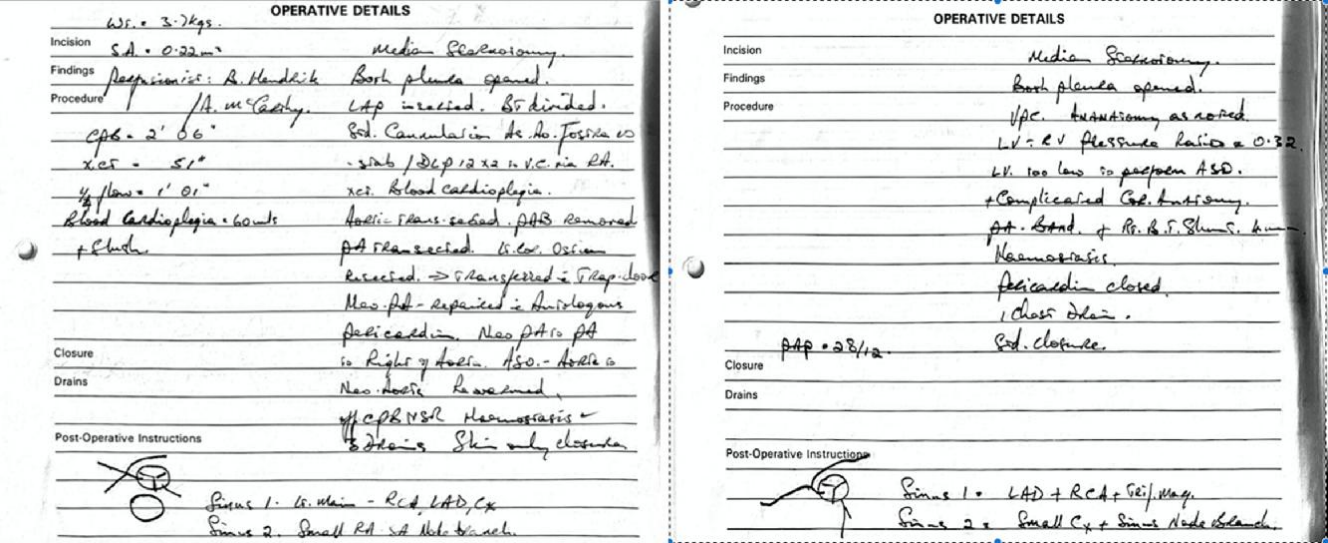
Excerpt from original operation in 1996 the detailing transfer of coronary arteries to current positions. *Source*: Mater Private Network.

The patient’s past surgical history was notable for a neonatal transposition of the great arteries requiring multiple surgeries including balloon atrial septostomy, pulmonary artery banding, Blalock-Taussig shunt, and ultimate arterial switch procedure which occurred within 2 weeks of birth. On careful scrutiny of the arterial switch operative notes, there was a description of a transfer of a ‘left coronary ostium’ thought to supply the right coronary artery (RCA), left anterior descending (LAD), and LCx from the native-aortic root to neo-aortic root ‘sinus 1’ and ‘sinoatrial (SA) node branch’ from the native-aortic root to ‘sinus 2’ (*Figure 1*). In hindsight, the LCx, with its aberrant origin, was likely overlooked and the nearby ‘SA node branch’ was instead transposed to the neo-aortic root leaving the LCx on the neo-pulmonary root and explaining the nearby surgical clips. He had no follow-up from an adult congenital heart disease (ACHD) team regarding this procedure. As a result, there were no previous investigations available for comparison. Subsequent discussion with the patient’s family practitioner elicited that routine follow-up had been offered but not availed of. His initial electrocardiogram (ECG) showed broad-complex ventricular tachycardia (BCT) at 235 bpm. The patient was haemodynamically stable at admission and conservative management was attempted with intravenous metoprolol 2.5 milligrams and intravenous amiodarone 300 milligrams over 30 min. This failed to resolve the arrythmia and external cardioversion was required. The subsequent in-ospital course involved an intensive care unit (ICU) admission for aspiration pneumonia related to an episode of vomiting on admission. Subsequently, the patient was transferred to our institution for ongoing cardiac management.

Blood work at the time of admission including electrolytes and cardiac enzymes were all within normal ranges, his troponin specifically <5. Baseline 12-lead ECG showed sinus rhythm with a normal QRS complex. His echocardiogram showed normal left ventricular (LV) dimensions, with subtle inferolateral hypokinesis and an ejection fraction (EF) of 45–50%.

**Figure 2:**
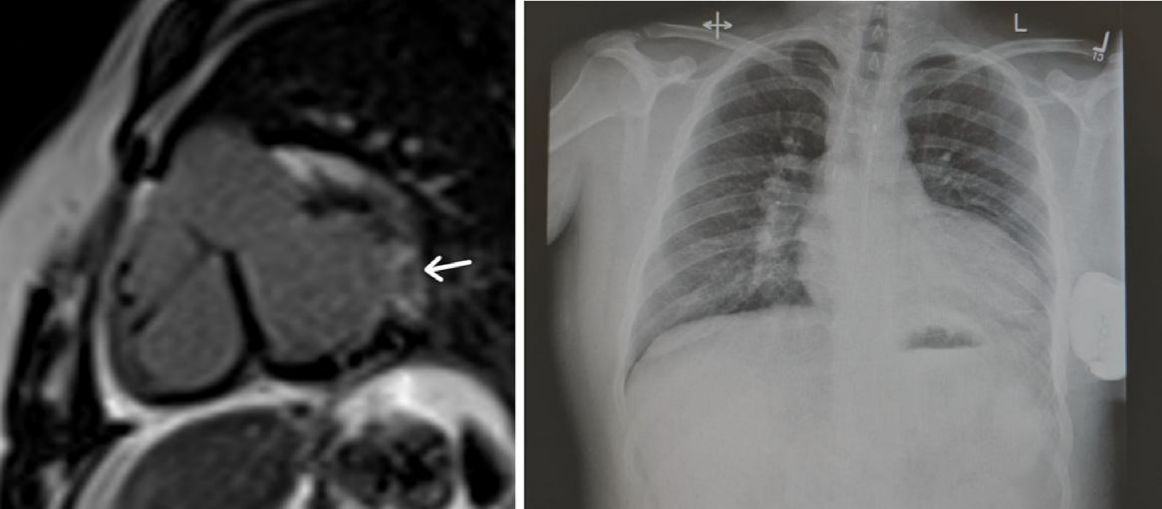
cardiac magnetic resonance [EF (50%)] showing late gadolinium enhancement in the basal anterolateral and inferolateral walls (white arrow) (left). Chest radiograph showing subcutaneous implantable cardioverter defibrillator insertion (right). *Source*: Mater Private Network.

Short axis Late gadolinium enhancement (LGE) cardiac magnetic resonance (CMR) imaging showed delayed enhancement in the basal anterolateral and inferolateral walls (*Figure 2*). There was a mildly dilated Left ventricle [LVEDVi 124 mL/m^2^ (60–112)] with low-normal systolic function (LVEF 50%). Normal right ventricular size and function.

**Figure 3:**
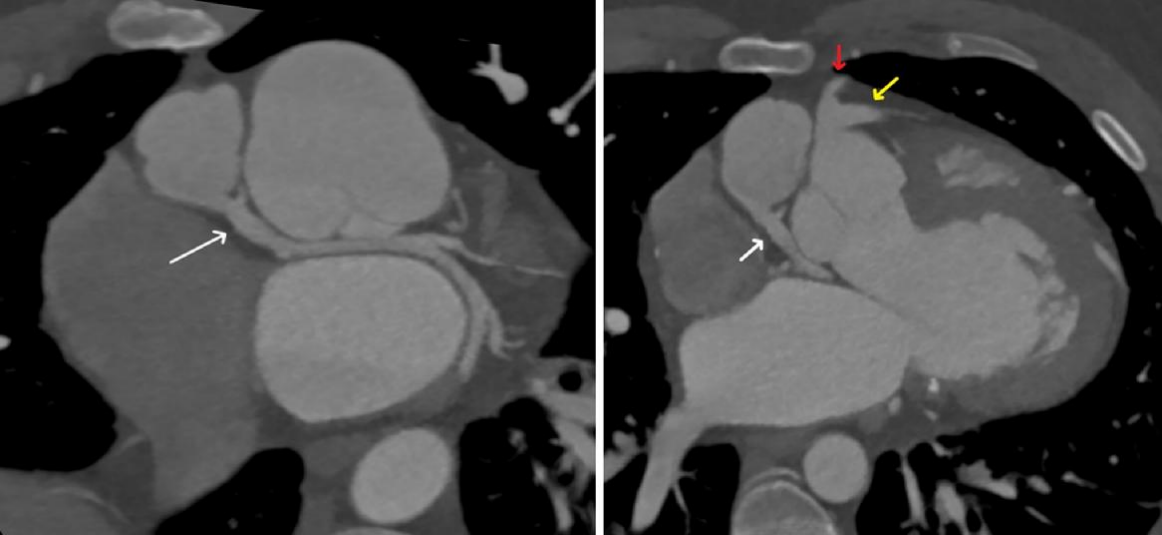
CT shows anomalous origin of the left circumflex artery arising from the pulmonary artery (left arrow) (left). CT demonstrating anomalous left circumflex artery as well as the right coronary artery ( top arrow) and left anterior descending (right arrow) arising from the right coronary cusp (right). *Source*: Mater Private Network.

Computed tomography coronary angiography (CTCA) showed a left main stem (LMS) arising from the right coronary cusp (RCC) and supplying the LAD (*Figure 3* yellow arrow) and RCA (*Figure 3* red arrow). LCx was seen arising from the main pulmonary artery (mPA) with co-located surgical clips (*Figure 3* white arrows). The LCx subtended the area of LGE on CMR imaging. Although a significant finding, given the patient’s history of cocaine and stimulant consumption this aberrant coronary artery anatomy was likely one of many inciting factors in this patient’s presentation.

**Figure 4:**
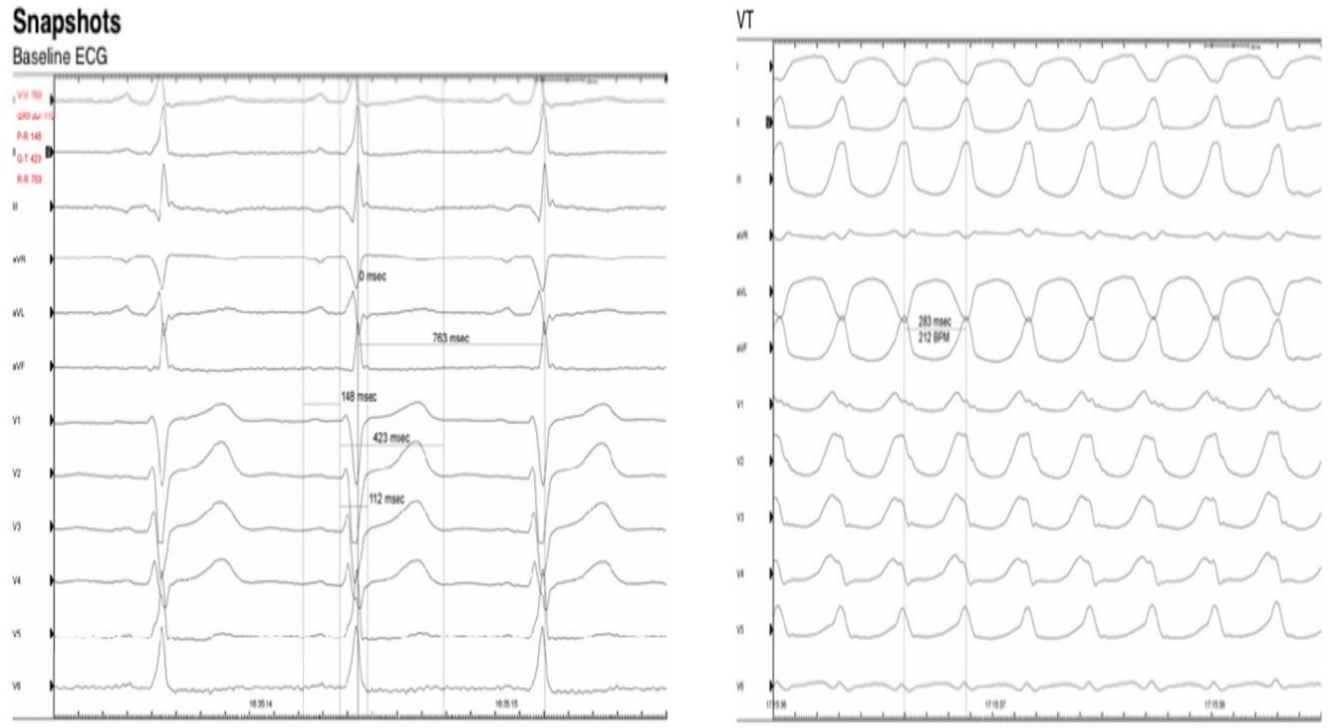
Electrophysiology study demonstrating inducible broad-complex tachycardia with double ventricular extrasystoles origin requiring termination. *Source*: Mater Private Network.

EPS with ventricular stimulation protocol unmasked a different morphology BCT to his original presenting arrhythmia at 210 bpm consistent with ventricular tachycardia (*Figure 4*), which required pace-termination.

**Figure 5:**
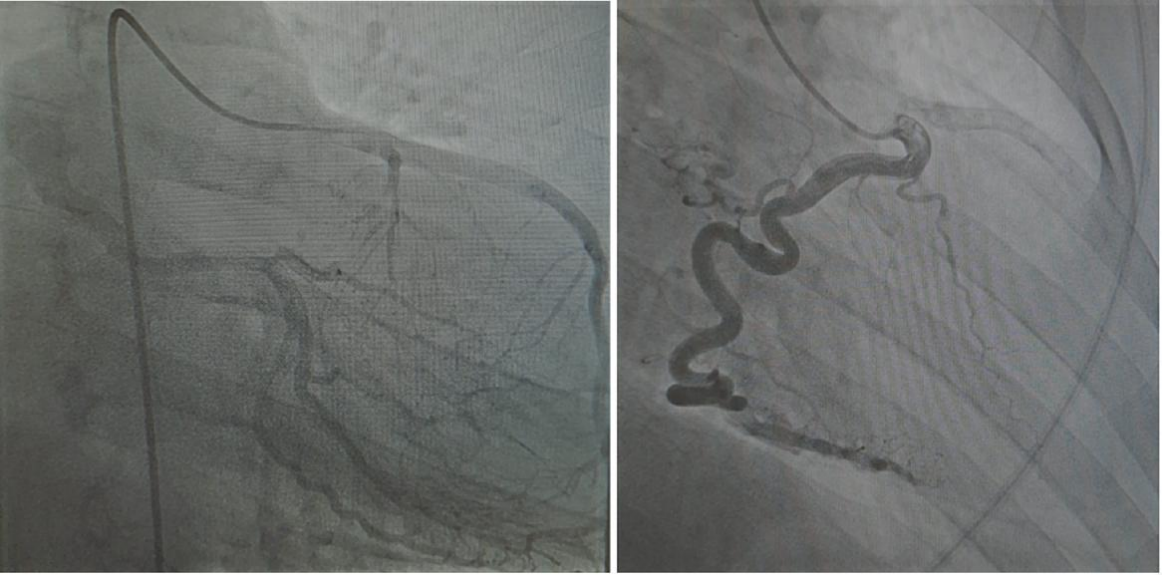
Anteroposterior caudal projection (CAU 20 deg) contrast opacifying left anterior descending, Retrogradely filling left circumflex artery, and emptying into the main pulmonary artery (left). Straight right anterior oblique (RAO) projection (RAO 30 deg). Catheter engaged and contrast opacifying right coronary artery predominantly. Ghosting of the left anterior descending (right). *Source*: Mater Private Network.

The patient was next listed for right-(RHC) and left heart catheterization (LHC) in order to his right heart pressures and to assess whether his ALCxAPA-supplied territory had enough pressure to maintain adequate blood supply to the systemic side of the heart. RHC showed mildly elevated right-sided pressures but no significant step-up or shunt in the mPA. LHC showed an anomalous LMS, LAD, and RCA arising from the right coronary cusp all widely patent, with collaterals to the LCx, which retrogradely filled to its os in the mPA. (*Figure 5*). A subcutaneous implantable cardioverter defibrillator (s-ICD) was next implanted.

S-ICD was implanted without issue and defibrillation thresholds were tested and satisfactory (*Figure 2*). S-ICD was chosen in lieu of a transvenous device due to the patient history of cocaine use which may predispose him to an increased risk of infective endocarditis. Ablation was not attempted in this patient due to a number of factors. First, the patient had experienced significant physiological stressors at the time of presentation including significant alcohol and cocaine intake followed by profuse vomiting and subsequent dehydration. Furthermore, our patient had not been on optimal medical therapy at the time of presentation and had no further episodes of arrhythmia since commencing 5 milligrams of once-daily bisoprolol. Finally, our patient had two separate sources of ventricular tachycardia. Posterolateral LV in origin in his local hospital and anterolateral LV origin during isoprenaline stimulated EPS. Following a discussion with the patient about the risks and benefits of ablation the decision was made to continue with medical therapy alone given his satisfactory response to this treatment.

Subsequent Multidisciplinary team (MDT) discussion determined the area of LGE on CMR imaging to be artefact given its size (∼25–50% transmural). As a result, this meeting determined the best course of action to be a focus on medical management with regular follow-up. As we will discuss, further comparison of both surgical and conservative management may be warranted to guide future decision-making with regard to this rare but serious anomalous coronary artery distribution.

## Discussion

ACHD has an estimated prevalence of 3000 cases per million people.^2^ Our patient is not strictly a case of congenital coronary artery anomaly in that his current coronary distribution is secondary to surgical intervention. Whether the long-term sequelae of surgically created ALCxAPA are similar to that of congenital ALCxAPA is not known and a literature review did not reveal any similar cases. Among patients with known deviation in normal coronary artery distribution, the LCx arising from the mPA is seldom seen. Ziermann *et al*. found in a review of 98 patients with anomalous origin of a coronary artery from the mPA the following distribution: 85.7% left coronary artery from the mPA, 6.1% LCx from the mPA, 4.1% RCA from the mPA, and 2.0% LAD from the mPA.^3^

Our patient presented with symptoms not necessarily indicative of ischaemia. Despite evidence of a previous myocardial infarction on imaging, the patient’s prevailing symptom was palpitations precipitated by an episode of ventricular flutter. This likely had been induced by myocardial stress in the setting of cocaine and stimulant consumption.^4^

Therefore the patient’s coronary artery anatomy may have predisposed him to developing symptoms in this setting although was likely not the sole inciting factor. Asymptomatic presentations while not uncommon are far from the rule in coronary artery distributions of this type. Guenther *et al*. found that while 23.9% of patients are diagnosed incidentally, the majority of asymptomatic presentations occur before the age of 20.^1,5^ While this data specifically concerns congenital ALCxAPA, it is not unreasonable to venture that our patient would follow a similar clinical course.

The exact anatomy of our patient’s coronary artery distribution was only elucidated following CMR and CT coronary angiography. This is not the first instance of MRI being used to diagnose this condition. Korosoglou *et al.* confirm that while conventional X-ray angiography may be used to diagnose aberrant coronary artery anatomy, CT and CMR imaging are often required to gain an unquestioned anatomical diagnosis of coronary artery origins.^6,7^

Interestingly, Guenther *et al*. also noted that while a number of congenital heart lesions and surgical repairs are associated with LCx originating from the mPA, transposition of the great vessels was not included. This is in keeping with our case of surgically created ALCxAPA and lends credence to the idea that this may be the only patient described in the literature with this surgical history and coronary artery anatomy.^1^

With regard to the management of this patient’s condition. Surgical intervention for anomalous coronary artery anatomy is the gold standard of care in both symptomatic patients and those demonstrating ischaemia on imaging.^8,9^ Data are understandably limited on outcomes and indications of this surgery given its rarity. The main options for treatment include aortic re-implantation, ligation, and coronary artery bypass graft (CABG) and ligation only. Guenther *et al* found that 82.6% of patients with the congenital ALCxAPA variant were offered some form of corrective surgery with the remainder being managed expectantly.^1^ Surgical outcomes for surgically created ALCxAPA have not thus far been studied likely due to low patient numbers. Other anomalous coronary artery variants such as those described in *Law et al.* found that overall operative mortality with regards to surgical re-implantation of anomalous RCA is low with 15 of 16 patients undergoing the procedure successfully and 14 being asymptomatic during a mean follow-up of 60.5 months.^5^ Eventually a MDT decision was made to forgo surgical re-implantation of the anomalous LCx. In part because the patient was now well-established on anti-remodelling medications with no therapies from his s-ICD, and in part owing to patient preference. However, the retrograde filling of the LCx to the mPA on angiography, along with LGE on CMR both hint at the potential to develop steal syndrome of the remaining viable coronary territories which could lead to scar formation and malignant ventricular arrhythmias. For our patient, the current plan is for annual stress perfusion CMR and surgery if any future evidence of new or expanded LGE.

In conclusion, further research will be required to determine whether the gold standard of treatment for ALCxAPA is a surgical intervention in all cases or whether medical management is appropriate in select patient groups.

## Data Availability

Data supporting the findings of this study is available from author (W.M.) upon reasonable request.

## Lead author biography

Dr. William Murray graduated with honours from Galway University medical school in 2022. He currently works at the Mater Misericordiae University Hospital. He has an interest in structural heart disease and cardiac electrophysiology.

**Consent:** The author/s confirms that written informed consent for submission and publication of this case report including image(s) and associated text has been obtained from the patient in line with COPE guidance.

**Funding:** None.

## References

[1] van der Bom T , Bouma BJ, Meijboom FJ, Zwinderman AH, Mulder BJ. The prevalence of adult congenital heart disease, results from a systematic review and evidence based calculation. Am Heart J 2012;164:568–575. 23067916 10.1016/j.ahj.2012.07.023

[2] Guenther TM , Sherazee EA, Gustafson JD, Wozniak CJ, Brothers J, Raff G. Anomalous origin of the circumflex or left anterior descending artery from the pulmonary artery. World J Pediatr Congenit Heart Surg 2020;11:765–775. 33164690 10.1177/2150135120938705

[3] Ziermann F HJ , Lange R, Ewert P, Hager A. Anomalous coronary arteries connected to pulmonary artery in combination with other congenital heart defects and extracardiac anomalies—overview of 98 patients. Cardiol Young 2017;27:S151.

[4] Winhusen T , Theobald J, Kaelber DC, Lewis D. The association between regular cocaine use, with and without tobacco co-use, and adverse cardiovascular and respiratory outcomes. Drug Alcohol Depend 2020;214:108136. 32623147 10.1016/j.drugalcdep.2020.108136 PMC7423623

[5] Law T , Dunne B, Stamp N, Ho KM, Andrews D. Surgical results and outcomes after reimplantation for the management of anomalous aortic origin of the right coronary artery. Ann Thorac Surg 2016;102:192–198. 27112655 10.1016/j.athoracsur.2016.02.002

[6] Korosoglou G , Ringwald G, Giannitsis E, Katus HA. Anomalous origin of the left circumflex coronary artery from the pulmonary artery. A very rare congenital anomaly in an adult patient diagnosed by cardiovascular magnetic resonance. J Cardiovasc Magn Reson 2008;10:4. 18272006 10.1186/1532-429X-10-4 PMC2244609

[7] Garcia CM , Chandler J, Russell R. Anomalous left circumflex coronary artery from the right pulmonary artery: first adult case report. Am Heart J 1992;123:526–528. 1736592 10.1016/0002-8703(92)90673-j

[8] Li D , Zhu Z, Zheng X, Wang Y, Wang Y, Xu R, et al Surgical treatment of anomalous left coronary artery from pulmonary artery in an adult. Coron Artery Dis 2015;26:723–725. 26180997 10.1097/MCA.0000000000000286 PMC4635871

[9] King NM , Tian DD, Munkholm-Larsen S, Buttar SN, Chow V, Yan T. The aberrant coronary artery—the management approach. Heart Lung Circ 2018;27:702–707. 28784571 10.1016/j.hlc.2017.06.719

